# The anticonvulsant retigabine is a subtype selective modulator of GABA_A_ receptors

**DOI:** 10.1111/epi.12950

**Published:** 2015-03-16

**Authors:** Marco Treven, Xaver Koenig, Elham Assadpour, Enkhbileg Gantumur, Christiane Meyer, Karlheinz Hilber, Stefan Boehm, Helmut Kubista

**Affiliations:** ^1^ Department of Neurophysiology and NeuropharmacologyCenter for Physiology and PharmacologyMedical University of ViennaViennaAustria

**Keywords:** Retigabine, Seizure‐like activity, Kv7 channels, Extrasynaptic GABA_A_ receptors

## Abstract

**Objective:**

Within its range of therapeutic plasma concentrations, the anticonvulsant retigabine (ezogabine) is believed to selectively act on Kv7 channels. Here, the contribution of specific γ‐aminobutyric acid (GABA)_A_ receptor subtypes to the antiseizure effects of retigabine was investigated.

**Methods:**

Using patch‐clamp recordings, seizure‐like activity, tonic currents, and GABA‐induced currents in hippocampal neurons were tested for their sensitivity toward retigabine, as were recombinant GABA_A_ receptors expressed in tsA 201 cells.

**Results:**

Retigabine reduced seizure‐like activity elicited by low Mg^2+^ in a concentration‐dependent manner with half maximal inhibition at 1 μm. Seizure‐like activity triggered by blocking either Kv7 channels or GABA_A_ receptors was equally reduced by retigabine, but when these channels/receptors were blocked simultaneously, the inhibition was lost. Retigabine (10 μm) enhanced bicuculline‐sensitive tonic currents in hippocampal neurons, but failed to affect GABA‐evoked currents. However, when receptors involved in phasic GABAergic inhibition were blocked by penicillin, retigabine did enhance GABA‐evoked currents. In tsA 201 cells expressing various combinations of GABA_A_ receptor subunits, 10 μm retigabine enhanced currents through α1β2δ, α4β2δ, α4β3δ, and α6β2δ receptors, but left currents through α1β2γ2S, α4β3γ2S, α5β3γ2S, and α6β2γ2S receptors unaltered. With αβ receptors, retigabine diminished currents through α1β2 and α4β3, but increased currents through α6β2 receptors. The enhancement of currents through α1β2δ receptors by retigabine was concentration dependent and became significant at 1 μm.

**Significance:**

These results demonstrate that retigabine is a subtype selective modulator of GABA_A_ receptors with preference for extrasynaptic δ‐containing receptors; this property may contribute to its broad antiepileptic effectiveness and explain its lack of effect on absence seizures.

Although an armamentarium of antiepileptic drugs has been available for half a century, seizures have remained uncontrolled in a considerable proportion of patients. Therefore, at least a dozen new antiepileptic drugs have been introduced within the past 20 years, many of which act via newly identified molecular targets.^1^ The avenue toward target identification with respect to antiepileptic pharmacotherapy is guided by the increasing knowledge regarding genetic epilepsies.^2^ Most of the mutations responsible for the development of seizures affect genes coding for various ion channels. Among such genes, the *KCNQ* family is a prominent group. *KCNQ* genes code for proteins that are pore‐forming subunits of Kv7 channels. In the nervous system, these channels activate at subthreshold potentials, give rise to noninactivating potassium currents, and thereby stabilize the membrane potential.^3^ Conceivably, loss‐of‐function mutations in these channels may lead to hyperexcitability of the affected neurons and thus to seizure‐like activity. Supporting this notion, activators of Kv7 channels have been developed to be used in conditions of neuronal hyperexcitability, such as pain and epilepsy.^4^ In the 1990s, retigabine, also known as ezogabine, had been found to exert potent anticonvulsant activity in a broad range of seizure models and to activate K^+^ channels in neurons and neuron‐like cells.^5^ In 2011, retigabine was finally approved for the treatment of partial seizures in adults^6^ and is now viewed as the first‐in‐class antiepileptic K^+^ channel opener.^7^

Mutations in Kv7 channels have been identified as the molecular basis for benign familial neonatal seizures (BFNS).^8^ These are multifocal tonic–clonic convulsions that typically emerge during the second or third postnatal day and disappear spontaneously after a few weeks or months.^2^ In light of this transient nature of seizures caused by mutations in Kv7 channels, it appears somewhat unexpected that an activator of these channels, such as retigabine, can provide sustained anticonvulsive activity. In fact, the spontaneous resolution of BFNS has been explained by the maturation of the central nervous system, in particular by the developmental switch of the γ‐aminobutyric acid (GABA)ergic system from excitatory to inhibitory.^8^ The inhibitory control of neuronal output by GABAergic neurons plays a pivotal role in orchestrating neuronal circuits to properly fulfil their physiologic functions,^9^ and potentiation of the GABAergic neurotransmission is the oldest mechanism of action known for antiepileptic drugs.^1^

GABAergic inhibition involves GABA_A_ and GABA_B_ receptors, the former being pentameric ligand‐gated ion channels composed of 1–4 different types of subunits of a repertoire of at least 19 proteins.^10^ When activated by GABA, these receptors conduct currents carried by Cl^−^ and ${\text{HCO}}_{3}^{-}$ ions, and such currents present themselves in two fundamentally different ways, as phasic and tonic currents, respectively. These different types of currents are mediated by separate sets of GABA_A_ receptors, namely synaptic and extrasynaptic receptors, which are characterized by distinct molecular architectures; synaptic receptors contain γ subunits, whereas most extrasynaptic receptors integrate δ subunits instead.^11^, ^12^

Retigabine opens Kv7 channels at concentrations of 1–6 μm.^7^ At 10 μm or above, retigabine has also been found to affect GABA_A_ receptors,^13^, ^14^ but this action is considered to be irrelevant with respect to its anticonvulsant effectiveness.^7^ Moreover, it is not known whether retigabine might differentiate between synaptic and extrasynaptic GABA_A_ receptors. This study uses an in vitro model of seizure‐like activity to reveal that retigabine's anticonvulsive activity involves Kv7 channels as well as GABA_A_ receptors and demonstrates that therapeutic concentrations of this drug act selectively on a subset of extrasynaptic GABA_A_ receptors.

## Methods

### Cell cultures and transfections

Primary cultures of hippocampal neurons were prepared as described in detail before.^15^ Tissue was obtained from Sprague‐Dawley rats, which were killed by decapitation in full accordance with all rules of the Austrian animal protection law (see http://www.ris.bka.gv.at/Dokumente/BgblAuth/BGBLA_2012_I_114/BGBLA_2012_I_114.pdf) and the Austrian animal experiment by‐laws (see http://www.ris.bka.gv.at/Dokumente/BgblAuth/BGBLA_2012_II_522/BGBLA_2012_II_522.pdf) which implement European (DIRECTIVE 2010/63/EU; see http://eur-lex.europa.eu/LexUriServ/LexUriServ.do?uri=OJ:L:2010:276:0033:0079:en:PDF) into Austrian law (all information accessed on July 2, 2014). The responsible animal welfare body is the “Ethics Committee of the Medical University of Vienna for Research Projects Involving Animals.” For the measurement of seizure‐like activity and tonic currents, cultures of hippocampal neurons were used >20 days after dissociation to ensure the establishment of fully functional synaptic contacts and thus proper neuronal networks.^15^ For GABA‐evoked currents, cultures were used after about 10 days.^16^

For heterologous expression of GABA_A_ receptors and Kv7 channels, tsA 201 cells (a subclone of human embryonic kidney 293 cells) were cultured in Dulbecco's modified Eagle's medium containing 1 g/L glucose and 10% heat‐inactivated fetal calf serum. Cells were transfected using ExGen 500 or Turbofect according to the manufacturer's recommendations, with a transfection ratio of 1:1 for αβ receptors, 1:1:8 for αβγ or αβδ receptors, and 1:1 for heteromeric Kv7.2/Kv7.3 channels. The day after transfection, cells were seeded at lower density into 35‐mm culture dishes and used for patch‐clamp recordings 24–48 h after transfection.

### Electrophysiology

All recordings were done at room temperature (20–24°C). Patch pipettes were made with a Sutter P97 horizontal puller (Sutter Instruments, Novato, CA, U.S.A.) using borosilicate glass capillaries (GB150‐8P; Science Products, Hofheim, Germany). Tip resistances were between 2 and 5 MΩ. Recordings were performed using the perforated patch method, with the exception of tonic currents for which the whole‐cell configuration was used instead. For perforated patch measurements, pipettes were front filled with internal solution and then back filled with the same solution containing 500 μg/mL amphotericin B. Recordings were started after 20–30 min when series resistance had dropped and stabilized below 20 MΩ.

Current clamp recordings of seizure‐like activity were performed using a Multiclamp 700B amplifier (Molecular Devices, Sunnyvale, CA, U.S.A.) as described before.^15^, ^17^ The internal (pipette) solution for current clamp recordings contained (mm): 120 potassium gluconate, 1.5 sodium gluconate, 3.5 NaCl, 1.5 CaCl_2_, 0.25 MgCl_2_, 10 2‐(4‐(2‐hydroxyethyl)‐1‐piperazinyl)‐ethansulfonic acid (HEPES), 10 glucose, and 5 ethylene glycol tetraacetic acid (EGTA) (pH was adjusted to 7.3 with KOH). The external bathing solution consisted of (mm): 140 NaCl, 3 KCl, 2 CaCl_2_, 2 MgCl_2_, 10 HEPES, and 20 glucose (pH was adjusted to 7.4 with NaOH). For low Mg^2+^ conditions, MgCl_2_ was omitted from the external solution. Alternatively, 30 μm XE991 and/or 30 μm bicuculline methiodide was added to induce seizure‐like activity.

For voltage clamp recordings, an Axopatch 200B amplifier was used.^16^ For the determination of tonic currents, the internal solution was composed of (mm): 135 CsCl, 10 HEPES, 10 EGTA, and 1 MgCl_2_ (adjusted to pH 7.3 with CsOH). For GABA‐evoked currents, the internal solution contained (mm): 140 KCl, 2 CaCl_2_, 0.7 MgCl_2_, 10 EGTA, and 10 HEPES (pH was adjusted to 7.3 with KOH). For currents through Kv7 channels,^16^ the internal solution was composed of (mm): 75 K_2_SO_4_, 55 KCl, 8 MgCl_2_, and 10 HEPES (adjusted to pH 7.3 with KOH). In all voltage clamp recordings, the external solution consisted of (mm): 140 NaCl, 20 glucose, 10 HEPES, 2.5 CaCl_2_, 2 MgCl_2_, and 3 KOH (pH was adjusted to 7.4 with NaOH). For the recording of tonic currents, 6‐cyano‐7‐nitroquinoxaline‐2,3‐dione (CNQX, 10 μm) and tetrodotoxin (1 μm) were added to suppress glutamatergic neurotransmission and action potential propagation, respectively.

For the recording of GABA‐evoked currents, cells were continuously superfused, and drugs were applied using a piezo‐switched fast‐step SF‐77B perfusion connected to an eight‐channel valve control VC‐8 System (Warner Instruments, Hamden, CT, U.S.A.). GABA currents were elicited by application of GABA for 3 s to cells clamped at −70 mV. Currents through Kv7 channels were elicited by depolarizing cells to −30 mV; once every 10 s, cells were hyperpolarized to −55 mV for 1 s periods to allow the channels to close and to observe the deactivation current during these hyperpolarizations, which is specific for the Kv7 channels;^18^ currents were quantified by measuring amplitudes observed at −30 mV.

### Data analysis and statistics

Seizure‐like activity in current clamp recordings involves enhanced discharge frequencies as well as depolarized membrane potentials and was thus quantified by determining area under the curve (AUC) values as described.^17^ Briefly, the area between the voltage trace and a baseline corresponding to the average resting membrane potential before onset of seizure‐like activity, was calculated (mV·msec) for periods of 90 s per condition. AUC values in the presence of retigabine were calculated as percentage of averaged AUC values obtained before and after the application of the drug. Amplitudes of tonic currents in voltage clamp recordings were averaged for periods of 3 s, and differences between amplitudes determined before and after the addition of bicuculline were calculated.

GABA‐evoked currents were assessed by means of peak amplitudes. For concentration response curves of GABA‐induced currents in the presence of either solvent or drugs, current amplitudes evoked by different GABA concentrations in solvent or drugs were normalized to that of a normalization current evoked by 100 or 300 μm GABA in solvent in the very same cell. To determine the effects of retigabine on currents evoked by a fixed GABA concentration, this selected GABA concentration was applied for 3 s in the continuous presence of either solvent or retigabine; amplitudes in the presence of solvent/retigabine were calculated as percentage of mean control GABA current amplitudes obtained before and after the application of solvent/retigabine, respectively. Statistical analysis and preparation of graphs was done with the GraphPad Prism 5.0 software (Graphpad Software Inc, La Jolla, CA, U. S. A). Concentration response data were fitted using the Hill equation. All values are presented as mean ± standard error of the mean (SEM), unless indicated otherwise. Significance levels are given as n.s. not significant, *p < 0.05, **p < 0.01, ***p < 0.001, ****p < 0.0001, with the statistical test and number of repetitions appropriately indicated in the text or figure legends.

### Drugs and materials

Rat GABA_A_ receptor subunit complementary DNAs (cDNAs) were generously provided by Werner Sieghart, Margot Ernst, and Petra Scholze (Center for Brain Research, Vienna, Austria),^19^ and plasmids for Kv7.2 and Kv7.3 channels by Mark Shapiro (San Antonio, TX, U.S.A.).^20^ Retigabine was obtained from Alomone (Jerusalem, Israel); tetrodotoxin (TTX) from Latoxan (Valence, France); GABA, kynurenic acid, XE 991, bicuculline methiodide, 6‐cyano‐7‐nitroquinoxaline‐2,3‐dione disodium salt (CNQX), putrescine, progesterone, poly‐d‐lysine, cytosine arabinoside, amphotericin B, as well as bulk chemicals from Sigma‐Aldrich (Vienna, Austria); and insulin, transferrin, and Na‐selenite from Roche (Mannheim, Germany). Dulbecco's modified Eagle's medium, Leibovitz L‐15 medium, penicillin, streptomycin and l‐glutamine were purchased from PAA Laboratories (Pasching, Austria). Papain was bought from Worthington (Lakewood, NJ, U.S.A.). Heat‐inactivated fetal calf serum was obtained from Invitrogen (Lofer, Austria). ExGen and Turbofect reagents were obtained from Fermentas (St. Leon‐Rot, Germany). Culture dishes were obtained from Nunc (Roskilde, Denmark).

## Results

### Suppression of seizure‐like activity by retigabine involves both Kv7 channels and GABA_A_ receptors

**Figure 1:**
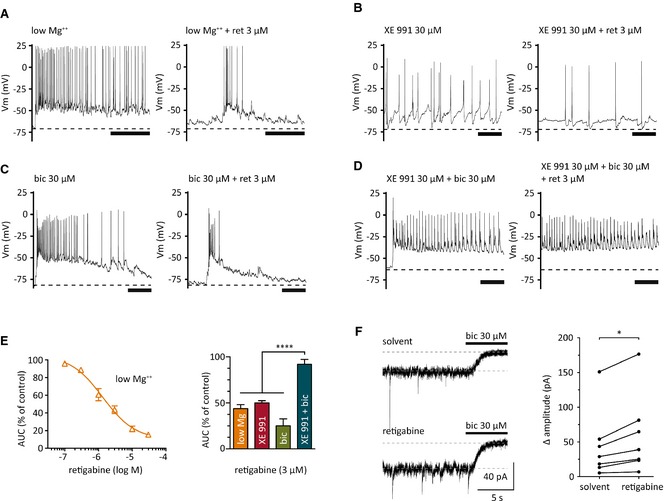
Effects of retigabine on seizure‐like activity and tonic currents in cultured hippocampal neurons. Seizure‐like activity was induced by low extracellular Mg^2+^ (Mg^2+^‐free solution), XE 991, or bicuculline (bic) applied either alone or together. (**A**–**D**) Original recordings of seizure‐like activity induced by low Mg^2+^ (**A**), 30 μm XE 991 (**B**), 30 μm bicuculline (bic; **C**), or XE 991 plus bicuculline (**D**). Traces were obtained before (left) and during (right) the presence of 3 μm retigabine (ret). Scale bars: 1 s. Dashed lines refer to the average membrane potential prior to the induction of seizure‐like activity. (**E**) Concentration–response curve for the inhibition of seizure‐like activity induced by low Mg^2+^ (orange) in the presence of the indicated concentrations of retigabine. This inhibition was evaluated by the reduction of the area under the curve (AUC), which was half maximal at 1.3 ± 0.3 μm (n = 7). The inhibitory effect of 3 μm retigabine was also quantified for seizure‐like activity induced by XE 991 (red), bicuculline (bic; green), or XE 991 plus bicuculline (green‐blue). AUC values obtained with XE 991 plus bicuculline were significantly different from all other values at p < 0.0001 (n = 7–9; one‐way analysis of variance [ANOVA] with Bonferroni's multiple comparison correction). (**F**) Holding currents recorded in one neuron at a potential of −70 mV in the presence of solvent, 10 μm retigabine, and 30 μm bicuculline (bic), respectively; bicuculline was first added in the presence of solvent and then in the presence of retigabine, as indicated by the bars. The graph shows differences in current amplitudes (Δ amplitude) measured before and after the addition of bicuculline for seven neurons; *p < 0.05 (Wilcoxon matched pairs signed‐rank test).

In rat hippocampal slices, the experimental paradigm of seizure‐like activity induced by low Mg^2+^ has been used before to provide evidence for the antiseizure activity of retigabine: 20–100 μm of the drug reduced seizure‐like events.^21^ This, however, is beyond therapeutic concentrations, which range up to 10 μm only.^22^ In primary cultures of dissociated rat hippocampi with well‐established synaptic contacts (i.e., at least 20 days in culture), low Mg^2+^ can also be used to trigger seizure‐like activity,^17^ and the latter was reduced by retigabine in a concentration‐dependent manner with half maximal inhibition at 1 μm (Fig. 1A,E).

The anticonvulsive action of retigabine is assumed to be based on its ability to open Kv7 channels.^7^ Despite this posit, the inhibitory effect of 3 μm retigabine remained unchanged when seizure‐like activity was induced by 30 μm XE 991 instead of low Mg^2+^ (Fig. 1B,E), the former being an irreversible inhibitor of Kv7 channels.^23^ To prove that the effect of retigabine on seizure‐like activity induced by XE 991 could not be mediated by an action on Kv7 channels, the combination of these two agents was tested on recombinant Kv7.2/Kv7.3 channel heteromers expressed in tsA 201 cells. The noninactivating currents through these channels determined at a potential of −30 mV were enhanced reversibly in the presence of 10 μm retigabine to 153.6 ± 7.3% of control values (n = 14). The subsequent application of 10 μm XE 991 reduced these current amplitudes to 5.0 ± 1.1% of control (n = 14; p < 0.05; analysis of variance followed by Dunn's multiple comparison test). Thereafter, the addition of retigabine (10 μm) to XE 991 left current amplitudes unchanged (5.1 ± 1.2% of control; n = 14; p > 0.05). After removal of both drugs, current amplitudes also remained suppressed (7.2 ± 1.2% of control; n = 13). Thus, in the presence of XE 991, retigabine is unable to open Kv7 channels. Therefore, its action on seizure‐like activity must have involved alternative mechanisms.

As high concentrations (>10 μm) of retigabine had been reported to act on GABA_A_ receptors,^13^, ^14^ the GABA_A_ receptor antagonist bicuculline methiodide was used at saturating concentrations (30 μm)^24^ to trigger seizure‐like activity. Three micromolar retigabine reduced seizure‐like activity caused by bicuculline methiodide to the same extent as seizure‐like activity triggered by either low Mg^2+^ or XE 991 (Fig. 1C,E). However, when seizure‐like activity was induced by XE 991 plus bicuculline, the inhibitory action of 3 μm retigabine was lost (Fig. 1D,E). Thus, the antiseizure activity of retigabine appears to involve both Kv7 channels and GABA_A_ receptors.

### Retigabine enhances bicuculline‐sensitive tonic currents

In voltage clamp recordings in cultures that displayed seizure‐like activity, retigabine (10 μm) was found to shift holding currents determined at −70 mV (not shown). To reveal whether such an effect might also occur independently of Kv7 channels, recordings were performed using the whole cell configuration with intracellular Cs^+^ to block K^+^ channels. Under these conditions, 10 μm retigabine enhanced standing inward currents (Fig. 1F). Such tonic currents involve continuous activity of extrasynaptic GABA_A_ receptors, which can be blocked by bicuculline.^25^ Here, bicuculline reduced amplitudes of tonic inward currents as expected (Fig. 1D). However, differences in tonic current amplitudes caused by 30 μm bicuculline were significantly larger in the presence of retigabine (59.7 ± 21.7 pA) than in the presence of solvent (45.0 ± 18.8 pA; p < 0.05 Wilcoxon matched pairs signed‐rank test). Thus, bicuculline‐sensitive tonic currents were enhanced by retigabine.

### Blockage of receptors involved in phasic GABAergic inhibition reveals an effect of low retigabine concentrations on GABA_A_ receptors

**Figure 2:**
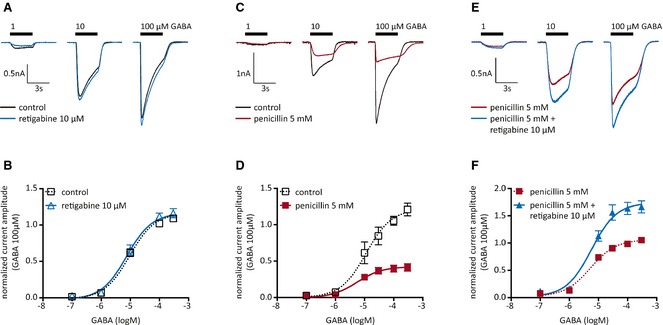
Effects of retigabine on GABA‐evoked currents in cultured hippocampal neurons. Currents were evoked by the application of the indicated concentrations of GABA in either solvent (control) or 5 mm penicillin, 10 μm retigabine, or both. (**A**) Original traces of currents evoked by the indicated concentrations of GABA in one hippocampal neuron in the presence of solvent (control; black) or 10 μm retigabine (blue). (**B**) Concentration–response curves for GABA‐evoked currents in the presence of solvent (control; black) or 10 μm retigabine (blue; n = 7). All peak current amplitudes determined in one neuron were normalized to the amplitude of the current triggered by 100 μm GABA in the presence of solvent. (**C**) Original traces of currents evoked by the indicated concentrations of GABA in one hippocampal neuron in the presence of solvent (control: black) or 5 mm penicillin (red). (**D**) Concentration response curves for GABA‐evoked currents in the presence of solvent (control; black) or 5 mm penicillin (red; n = 5). All peak current amplitudes determined in one neuron were normalized to the amplitude of the current triggered by 100 μm GABA in the presence of solvent. (**E**) Original traces of currents were evoked by the indicated concentrations of GABA in one hippocampal neuron in the presence of either 5 mm penicillin (red) or 5 mm penicillin plus 10 μm retigabine (blue). (**F**) Concentration–response curves for GABA‐evoked currents in the presence of either 5 mm penicillin (red) or 5 mm penicillin plus 10 μm retigabine (blue; n = 7). All peak current amplitudes determined in one neuron were normalized to the amplitude of the current triggered by 100 μm GABA in the presence of penicillin only. Maximal GABA current amplitudes were significantly larger in the presence of retigabine (p < 0.0001), whereas EC_50_ values remained unchanged (p > 0.8; F test, n = 7).

At concentrations of 10 μm and above, retigabine has been found to enhance inhibitory postsynaptic currents^14^ as well as GABA‐evoked currents^13^ in cortical neurons. In hippocampal neurons lacking functional synaptic GABAergic input, as used here, GABA‐induced currents were not affected by 10 μm retigabine (Fig. 2A,B). In hippocampal neurons, 5 mm penicillin has been shown to block phasic GABAergic inhibition without affecting tonic inhibition.^26^ Here, 5 mm penicillin reduced GABA‐induced currents in a noncompetitive manner (Fig. 2C,D). Moreover, GABA‐evoked currents in the presence of penicillin were enhanced by 10 μm retigabine (Fig. 2E,F). Thus, retigabine exerted a facilitatory effect on GABA_A_ receptors when receptors involved in phasic GABAergic inhibition were blocked.

At low GABA concentrations, current amplitudes are very small (Fig. 2F) and easily biased by time‐dependent changes in experimental conditions as well as by eventual minute contaminations by saturating GABA concentrations. Therefore, in additional experiments we specifically tested for the effects of 10 μm retigabine at low GABA concentrations; 1 μm GABA was applied in the presence of 5 mm penicillin plus solvent and of 5 mm penicillin plus 10 μm retigabine, respectively. Amplitudes in the presence of 10 μm retigabine (129.7 ± 3.7% of control; n = 7) were significantly larger than those in the presence of solvent (102.1 ± 3.4% of control; n = 7; p < 0.01; paired *t*‐test subsequent to a Kolmogorov‐Smirnov test).

### Retigabine preferentially acts on GABA_A_ receptors containing δ subunits

**Figure 3:**
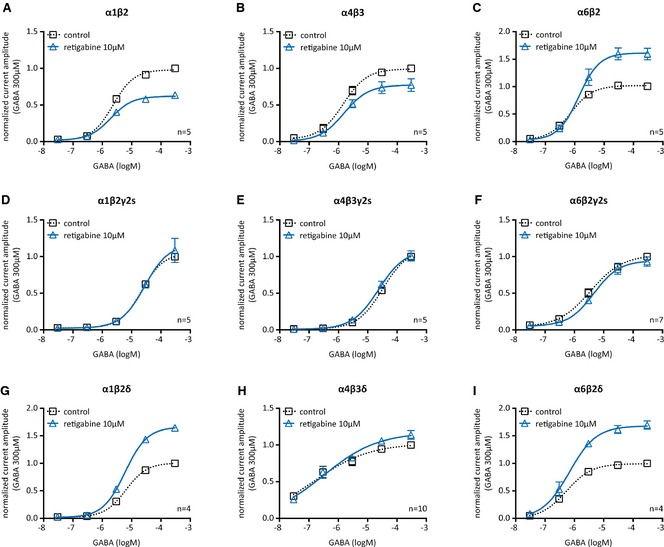
Effects of retigabine on GABA‐evoked currents in tsA 201 cells expressing various combinations of GABA_A_ receptor subunits. Currents through α1β2 (**A**), α4β3 (**B**), α6β2 (**C**), α1β2γ2S (**D**), α4β3γ2S (**E**), α6β2γ2S (**F**), α1β2δ (**G**), α4β3δ (**H**), and α6β2δ (**I**) receptors were evoked by the application of the indicated concentrations of GABA in the presence of solvent (control; black) or 10 μm retigabine (blue). For original sample traces see Figure 4A,C. For the concentration–response curves, all peak current amplitudes determined in one cell were normalized to the amplitude of the current triggered by 300 μm GABA in the presence of solvent. Data were fitted to a Hill equation; parameters are given in Table 1.

Tonic GABAergic inhibition is mediated by extrasynaptic GABA_A_ receptors, which do not contain γ subunits, but most frequently δ.^11^, ^12^ To explore a potential subtype selective action of retigabine, several subunit combinations typical for synaptic (α1β2γ2S) and extrasynaptic (α1β2δ, α4β3δ, α5β3γ2S, and α6β2δ) GABA_A_ receptors were expressed in tsA 201 cells and currents through these receptors were determined. Retigabine (10 μm) enhanced the currents through the δ‐containing receptors, but left the currents through the γ‐containing receptors unaltered (Fig. 3D,G–I; Table 1). To investigate this apparent subtype selectivity in a more systematic manner, the δ subunits in α1β2δ, α4β3δ, and α6β2δ were replaced by γ2S. Consistently, retigabine only potentiated δ (Fig. 3G–I), but not γ2S‐containing receptors (Fig. 3D–F). For comparison, the above combinations of α and β subunits were also expressed without either γ2S or δ subunits. In these receptors, retigabine (10 μm) caused an inhibition of α1β2 and α4β3, but a facilitation of α6β2 (Fig. 3A–C). With all these receptors, retigabine affected only the maxima of the concentration response curves for GABA‐induced currents, but left the concentrations required for half maximal current activation (EC_50_) unaltered (Table 1). To reveal whether the type of β subunit incorporated into the receptors also plays a role, the effect of 10 μm retigabine was compared for α4β2δ and α4β3δ, but no significant differences were observed (Table 1).

**Table 1:** Hill equation parameters for GABA concentration–response relations of currents through various recombinant GABA_A_ receptors in the presence of either solvent or 10 μm retigabine

Isoform	Solvent		Retigabine (10 μm)	
	EC_50_ (μm)	E_max_ (normalized)	EC_50_ (μm)	E_max_ (normalized)
α1β2	2.30 ± 0.19	0.98 ± 0.02	1.93 ± 0.22 n.s.	0.62 ± 0.01***
α1β2γ2S	22.19 ± 2.19	1.05 ± 0.03	27.92 ± 10.91 n.s.	1.17 ± 0.16 n.s.
α1β2δ	6.37 ± 0.82	1.01 ± 0.03	6.01 ± 0.67 n.s.	1.66 ± 0.04***
α4β3	1.45 ± 0.33	0.99 ± 0.03	1.55 ± 0.65 n.s.	0.77 ± 0.05*
α4β3γ2S	32.05 ± 3.22	1.10 ± 0.04	23.52 ± 5.54 n.s.	1.09 ± 0.08 n.s.
α4β3δ	0.16 ± 0.07	1.02 ± 0.06	0.32 ± 0.14 n.s.	1.17 ± 0.07*
α4β2δ	1.43 ± 0.13	1.00 ± 0.01	1.26 ± 0.25 n.s.	1.19 ± 0.04**
α5β3γ2S	5.66 ± 1.41	1.04 ± 0.06	5.57 ± 1.76 n.s.	0.97 ± 0.07 n.s.
α6β2	0.74 ± 0.13	1.02 ± 0.02	1.37 ± 0.44 n.s.	1.61 ± 0.08**
α6β2γ2S	3.55 ± 0.81	1.02 ± 0.05	4.60 ± 1.31 n.s.	0.95 ± 0.06 n.s.
α6β2δ	0.53 ± 0.02	0.99 ± 0.02	0.67 ± 0.19 n.s.	1.68 ± 0.07***

Concentration–response curves of currents through the listed GABA_A_‐receptor isoforms were obtained in the presence of either solvent or 10 μm retigabine (F test; n = 4–10; n.s. = no significant difference, *p < 0.05, **p < 0.01, ***p < 0.001 vs. the corresponding values in the presence of solvent). Representative concentration–response curves are displayed in Figure 3.

To specifically test for the effects of 10 μm retigabine on currents evoked by low GABA concentrations in the recombinant receptors, 1 μm GABA was applied to α1β2δ and α4β3δ receptors in the continuous presence of either solvent or retigabine. As with the native receptors above, amplitudes in the presence of 10 μm retigabine (α1β2δ: 125.9 ± 9.0% of control; n = 5; α4β3δ: 119.5 ± 8.0% of control; n = 6) were significantly larger than those in the presence of solvent (α1β2δ: 100.3 ± 0.7% of control; n = 5; p < 0.01; α4β3δ: 99.1 ± 1.6% of control; n = 6; p < 0.05; paired *t*‐test subsequent to a Kolmogorov‐Smirnov test).

### Concentration dependence of the effects of retigabine on GABA_A_ receptors

**Figure 4:**
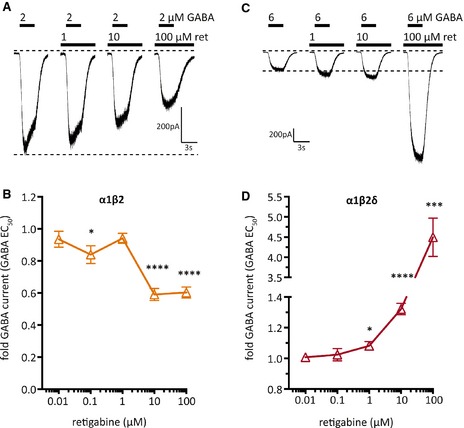
Retigabine concentration–response relation for GABA‐evoked currents through α1β2 or α1β2δ GABA_A_ receptors expressed in tsA 201 cells. Currents were evoked by the application of either 2 μm (**A** and **B**) or 6 μm (**C** and **D**) GABA (the EC_50_ values for α1β2 and α1β2δ receptors, respectively; see Table 1), in the presence of either solvent or the indicated concentrations of retigabine (ret). (**A**,** C**) Original traces of currents evoked by GABA in a cell expressing either α1β2 (**A**) or α1β2δ (**C**). (**B**,** D**) Concentration–response curves for the effect of retigabine on GABA‐induced currents in cells expressing either α1β2 (**B**; orange) or α1β2δ (**D**; red). Peak current amplitudes determined in the presence of the indicated concentrations of retigabine were normalized to the amplitudes obtained in the presence of solvent (n = 8). *, ***, **** indicate significant differences versus the amplitudes obtained in the presence of solvent at p < 0.05, p < 0.001, and p < 0.0001, respectively.

To reveal in which concentration range retigabine may affect the GABA_A_ subunit combinations described earlier, currents through α1β2 and α1β2δ receptors were triggered by GABA concentrations causing half maximal current amplitudes (i.e., 2 and 6 μm, respectively) in the presence of different retigabine concentrations. A significant increase in the amplitudes of currents through the α1β2δ receptor was found at concentrations as low as 1 μm (Fig. 4C,D). For the inhibition of currents through α1β2 receptors, higher concentrations were required (Fig. 4A,B).

## Discussion and Conclusions

Opening of neuronal Kv7 channels is regarded the sole mechanism of action of the novel antiepileptic drug retigabine.^4^, ^7^ Its anticonvulsive activity was confirmed using the well‐established in vitro paradigm of seizure‐like activity induced by low Mg^2+^. In these experiments, retigabine reduced neuronal firing in a concentration‐dependent manner with half maximal effects at about 1 μm. This drug is known to cause half‐maximal activation of homo‐ as well as heteromeric Kv7 channels in a similar concentration range.^7^ Unexpectedly, this inhibitory effect of retigabine was the same when seizure‐like activity was triggered by blocking Kv7 channels with XE 991. In addition, seizure‐like activity induced by the GABA_A_ receptor antagonist bicuculline was reduced to the same extent by retigabine. However, when Kv7 channels and GABA_A_ receptors were blocked simultaneously, the inhibitory action of retigabine was lost. Moreover, retigabine (10 μm) enhanced tonic currents when K^+^ channels were blocked by intracellular Cs^+^, and this effect was bicuculline sensitive. These results suggest GABA_A_ receptors as alternative therapeutic targets for retigabine and led us to investigate the interactions between these receptors and the antiepileptic drug in detail.

Previously, retigabine has been reported to increase the amplitudes and to prolong the decay of inhibitory postsynaptic currents in mouse cortical neurons.^14^ Retigabine has also been shown to reduce GABA release from rat hippocampal synaptosomes,^27^ but this latter action appeared to lack in the study on mouse cortical neurons.^14^ In any case, effects of retigabine on GABAergic neurotransmission may involve presynaptic as well as postsynaptic effects, the former ones being most likely mediated by an action on Kv7 channels.^27^ In the present study, confounding effects arising at a presynaptic site of action were avoided by the two following measures: (1) by activating the receptors through exogenous application of GABA and (2) by using hippocampal neurons lacking functional synaptic GABAergic input. Under these conditions, 10 μm retigabine did not affect currents through GABA_A_ receptors.

In cultured hippocampal neurons, GABAergic inhibition occurs in two major forms, phasic and tonic inhibition, and the former can be prevented by penicillin.^26^ When receptors involved in phasic inhibition were blocked by penicillin, GABA‐evoked currents were potentiated by 10 μm retigabine. Thus, retigabine appears to selectively act on receptors mediating tonic GABAergic inhibition. Phasic GABAergic inhibition is mediated by synaptic GABA_A_ receptors, tonic inhibition by extrasynaptic ones; the latter contain mostly δ instead of γ subunits.^11^, ^12^ Despite this disparity in receptor composition, the differential effect of penicillin on phasic and tonic GABAergic inhibition^26^ is not due only to subunit specificity of the antibiotic. Even though penicillin causes less inhibition in δ‐containing than in γ2‐containing receptors, the preferential block of phasic inhibition is also related to the conditions of receptor activation: currents evoked by high GABA concentrations are reduced to a greater extent than currents elicited by lower concentrations, and peak current amplitudes are efficiently diminished, whereas steady state current levels are hardly affected.^28^ Thus, the results obtained in hippocampal neurons in the presence of penicillin do not permit unequivocal conclusions regarding the GABA_A_ receptor subtype preference of retigabine.

The most prevailing extrasynaptic GABA_A_ receptors are composed of the following subunits: α1βδ in hippocampal and cortical interneurons; α4βδ in thalamic relay neurons, hippocampal dentate granule cells, and cortical pyramidal neurons in addition to medium spiny neurons in the striatum; α6β2δ in cerebellar granule cells; and α5βγ2 in hippocampal and cortical pyramidal neurons.^11^, ^12^ Previously, a structural analogue of retigabine (AA29504) has been found to display differing actions at α1β3γ2S and α4β3δ receptors.^29^ In the present study, retigabine enhanced currents through all recombinant GABA_A_ subunit combinations containing δ, but left currents through all the tested γ2S‐containing receptors unaltered. In particular, α1β2δ, α4β2/3δ, and α6β2δ were potentiated by retigabine, and the γ2S‐containing counterparts were not affected. Some of the extrasynaptic GABA_A_ receptors in hippocampal neurons may consist of αβ subunits only.^30^ With receptors containing solely αβ subunits, the effect of retigabine appeared to depend on the α subunit: currents through α1β2 and α4β3 receptors were reduced, whereas those through α6β2 were enhanced by the antiepileptic drug. Because α6 subunits are not present in the hippocampus,^31^ the enhancement of GABA‐evoked currents in the cultured neurons is most likely due to the facilitatory effect on δ‐containing receptors.

At recombinant Kv7 channels, retigabine concentrations required for half maximal current enhancement lie in the low micromolar range.^7^ When widely varying concentrations of retigabine were tested for their effects on recombinant GABA_A_ receptors, a statistically significant enhancement of currents through δ‐containing receptors was found at concentrations as low as 1 μm. High retigabine concentrations led to a dramatic current enhancement of more than fourfold, but a saturation of the effect was not reached, which prevents a reliable calculation of values for half maximal effects. In pharmacokinetic studies, retigabine plasma concentrations in humans reached 5 μm.^32^ Similar concentrations were also achieved in clinical trials.^22^ Given that the plasma protein binding of the drug amounts to about 80%, free plasma concentrations can be expected to be around 1 μm.^22^ Although levels of retigabine in the human brain remain unknown, the drug has been shown to accumulate in the brain of rodents. This cerebral accumulation was dose dependent and more than sixfold in comparison with plasma concentrations, when doses of ≥2 mg per kg body weight (corresponding to human doses of >150 mg) had been applied to rats.^33^ Thus, therapeutic levels of retigabine in the brain can be expected to reach values as high as 10 μm and to be sufficient to modulate Kv7 channels as well as δ‐containing GABA_A_ receptors.

The perception of δ‐containing GABA_A_ receptors as therapeutic targets for retigabine leads to the pathophysiology of such receptors in epilepsy. In various models of temporal lobe epilepsy, δ subunit expression and functions of extrasynaptic GABA_A_ receptors undergo complex changes.^34^, ^35^ Loss of neurosteroids that activate δ‐containing GABA_A_ receptors increases seizure frequency,^34^ whereas overexpression and activation of extrasynaptic GABA_A_ receptors reduces seizure‐like activity in vitro and in vivo, respectively.^36^ Extrasynaptic receptors display high affinities for GABA and are activated by low ambient GABA concentrations.^12^ These latter concentrations are controlled by GABA reuptake and metabolism, and these two mechanisms are suppressed by tiagabine and vigabatrin, respectively, two antiepileptic drugs in clinical use for about two decades.^1^ Although these agents lead to an activation of extrasynaptic GABA_A_ receptors indirectly through an increase in GABA concentrations, one of the most recently developed antiepileptic drugs, ganaxolone, directly activates the receptors and prefers δ containing over other types of GABA_A_ receptors.^37^ Hence, preferential activation of δ‐containing GABA_A_ receptors is an established mechanism of action in antiepileptic pharmacotherapy. This, however, does not hold true for all types of seizures. Enhanced activity of extrasynaptic GABA_A_ receptors in thalamocortical neurons is a common pathophysiologic mechanism in several models of absence epilepsy,^38^ and absence seizures are exacerbated by drugs that activate δ‐containing GABA_A_ receptors.^35^, ^38^ Retigabine has been found to be effective in virtually all types of seizure models, with the notable exception of absence seizures, even though Kv7 channels are highly expressed in the thalamus.^22^ Thus, the selective action of retigabine on δ‐containing versus other GABA_A_ receptors may not only contribute to its broad spectrum of antiepileptic effectiveness, but also explain its hitherto inexplicable lack of effect in absence seizures.

## Disclosure

None of the authors has any conflict of interest to disclose. We confirm that we have read the Journal's position on issues involved in ethical publication and affirm that this report is consistent with those guidelines.
